# Assessing the effect of nitisinone induced hypertyrosinaemia on monoamine neurotransmitters in brain tissue from a murine model of alkaptonuria using mass spectrometry imaging

**DOI:** 10.1007/s11306-019-1531-4

**Published:** 2019-04-29

**Authors:** A. S. Davison, N. Strittmatter, H. Sutherland, A. T. Hughes, J. Hughes, G. Bou-Gharios, A. M. Milan, R. J. A. Goodwin, L. R. Ranganath, J. A. Gallagher

**Affiliations:** 1 Department of Clinical Biochemistry and Metabolic Medicine, Liverpool Clinical Laboratories, Royal Liverpool University Hospitals Trust, Liverpool, L7 8XP UK; 2 Musculoskeletal Biology I, Institute of Ageing and Chronic Disease, University of Liverpool, Liverpool Health Partners, Liverpool, UK; 3 Pathology, Drug Safety and Metabolism, IMED Biotech Unit, AstraZeneca, Cambridge, UK

**Keywords:** Alkaptonuria, Neurotransmitter, Imaging, Mass spectrometry, Dopamine, Serotonin, Tyrosine, Tyramine, Tryptophan

## Abstract

**Objective:**

Nitisinone induced hypertyrosinaemia is a concern in patients with Alkaptonuria (AKU). It has been suggested that this may alter neurotransmitter metabolism, specifically dopamine and serotonin. Herein mass spectrometry imaging (MSI) is used for the direct measurement of 2,4-diphenyl-pyranylium tetrafluoroborate (DPP-TFB) derivatives of monoamine neurotransmitters in brain tissue from a murine model of AKU following treatment with nitisinone.

**Methods:**

Metabolite changes were assessed using MSI on DPP-TFB derivatised fresh frozen tissue sections directing analysis towards primary amine neurotransmitters. Matched tail bleed plasma samples were analysed using LC-MS/MS. Eighteen BALB/c mice were included in this study: HGD^−/−^ (n = 6, treated with nitisinone – 4 mg/L, in drinking water); HGD^−/−^ (n = 6, no treatment) and HGD^+/−^ (n = 6, no treatment).

**Results:**

Ion intensity and distribution of DPP-TFB derivatives in brain tissue for dopamine, 3-methoxytyramine, noradrenaline, tryptophan, serotonin, and glutamate were not significantly different following treatment with nitisinone in HGD^−/−^ mice, and no significant differences were observed between HGD^−/−^ and HGD^+/−^ mice that received no treatment. Tyrosine (10-fold in both comparisons, p = 0.003; [BALB/c HGD^−/−^ (n = 6) and BALB/c HGD^+/−^ (n = 6) (no treatment) vs. BALB/c HGD^−/−^ (n = 6, treated)] and tyramine (25-fold, p = 0.02; 32-fold, p = 0.02) increased significantly following treatment with nitisinone. Plasma tyrosine and homogentisic acid increased (9-fold, p = < 0.0001) and decreased (9-fold, p = 0.004), respectively in HGD^−/−^ mice treated with nitisinone.

**Conclusions:**

Monoamine neurotransmitters in brain tissue from a murine model of AKU did not change following treatment with nitisinone. These findings have significant implications for patients with AKU as they suggest monoamine neurotransmitters are not altered following treatment with nitisinone.

## Introduction

**Fig. 1:**
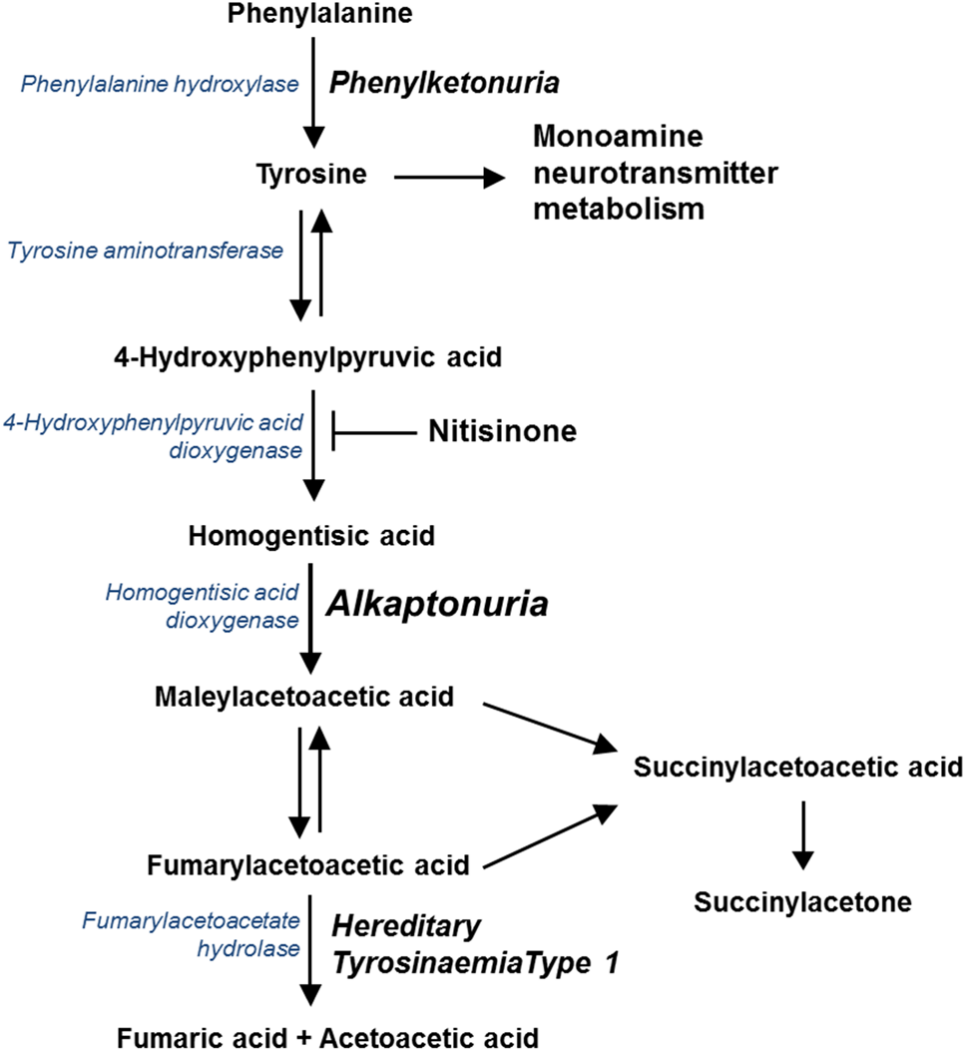
Tyrosine metabolic pathway—highlighting the site of the enzyme defect observed in Phenylketonuria (phenylalanine hydroxylase, *PAH* EC 1.14.16.1) Alkaptonuria (homogentisate dioxygenase, *HGD* EC 1.13.11.5) and Hereditary Tyrosinaemia Type 1 (maleylacetoacetate isomerase, *MAI* EC 5.2.1.2), and the site of action of nitisinone inhibiting 4-hydroxyphenylpyruvate dioxygenase (*HPPD* EC 1.13.11.27). Adapted from Davison et al. 2018b

Alkaptonuria (AKU, OMIM: 203500) is a rare bi-allelic autosomal recessive disorder of the tyrosine metabolic pathway, occurring 1 in 100,000–250,000 of the general population (Phornphutkul et al. 2002) and arises from a congenital deficiency in the enzyme homogentisate-1,2-dioxygenase (*HGD*, E.C.1.12.11.5) (Fig. 1). The biochemical hallmark of AKU is that circulating concentrations of homogentisic acid (HGA) markedly increase, and it is this that is thought to be responsible for many of the complications observed (for a detailed review see Ranganath et al. 2013). Treatment for this condition is largely based on supportive and palliative measures that include pain relief and anti-inflammatory medications, dietary protein restriction and joint replacement (Ranganath et al. 2013). Currently the drug nitisinone, a competitive inhibitor of hydroxyphenylpyruvic acid dioxygenase (*HPPD*, E.C. 1.13.11.27) (Fig. 1), is being evaluated as a potential treatment (Ranganath et al. 2016; Milan et al. 2017; SONIA-2—ClinicalTrials.gov Identifier: NCT01916382). This drug acts to move the metabolic block in the tyrosine metabolic pathway resulting in a marked reduction in the circulating concentration of HGA and a consequential significant increase in tyrosine, creating a so called ‘pseudo type-3 Tyrosinaemia’ picture (Suwannarat et al. 2005; Introne et al. 2011; Olsson et al. 2015; Ranganath et al. 2016; Milan et al. 2017; Davison et al. 2018a, b, c). Hypertyrosinaemia has also been well documented in patients with Hereditary Tyrosinaemia type-1 (HT1, OMIM 276700) that are treated with nitisinone (Lindstedt et al. 1992; Zeybek and Zubarioglu 2017; van Ginkel et al. 2017).

The metabolic fate of these supraphysiological tyrosine concentrations is unknown. It has been proposed in HT1 that hypertyrosinaemia may contribute to the neurodevelopmental delay that is frequently observed in children (McKiernan et al. 2015). Several mechanisms have been proposed for this, including increased transport of tyrosine into the brain; decreased transport of other neutral amino acids into the brain (specifically tryptophan); increased central nervous system dopamine; decreased central nervous system serotonin, oxidative damage from δ-aminolevulinic acid and succinylacetone or modification of neuronal proteins (Hillgartner et al. 2016; Thimm et al. 2011). It has also been suggested that altered serotonin metabolism may be due to direct inhibition of tryptophan hydroxylase (TPH; EC 1.14.16.4) activity by tyrosine, which leads to a reduced biosynthesis of serotonin (Thimm et al. 2011).

In AKU there is uncertainty about whether the hypertyrosinaemia may alter neurotransmitter metabolism and specifically whether this may lead to depression or altered cognition through the mechanisms detailed above. Recently Davison et al. (2018a) concluded that treatment with nitisinone is unlikely to cause depression in patients with AKU in a study that assessed urinary neurotransmitter metabolite concentrations, in a cohort of patients with AKU. This study did show an increase in urinary 3-methoxytyramine (3-MT, dopamine metabolite) and a decrease in urinary 5-hydroxyindole acetic acid (serotonin metabolite) following treatment. These changes did not correlate with Beck’s depression inventory-II scores. Similar biochemical findings were also reported in a study that evaluated different doses of nitisinone (0–8 mg daily for 4-weeks) in patients with AKU over a 4-week period (Davison et al. 2018b).

While previous studies have demonstrated that hypertyrosinaemia clearly results in altered peripheral metabolism of dopaminergic and serotoninergic neurotransmitter metabolites they are limited as they are not a direct reflection of neurotransmitter metabolism in the central nervous system, which is directly linked to mood and cognition. Direct measurement of neurotransmitter concentrations in cerebrospinal fluid and or in the brain are not feasible in this patient group owing to their complex musculoskeletal comorbidities.

Herein for the first time we report a direct approach to assess whether hypertyrosinaemia effects monoamine neurotransmitter metabolism. Desorption electrospray ionisation mass spectrometry imaging (DESI-MSI) was used to measure the ion intensity and distribution of 2,4-diphenyl-pyranylium tetrafluoroborate (DPP-TFB) derivatives of monoamine neurotransmitters in brain tissue from a murine model of AKU (BALB/c HGD^−/−^) (Preston et al. 2014) following treatment with nitisinone. Applying an imaging approach will enable us to identify whether any occurring changes are only appearing in certain brain substructures or are systemic to the whole brain. Chemical charge tagging of monoamine neurotransmitters (primary amines) with DPP-TFB was carried out as previous studies have demonstrated significant improvements in signal intensity compared to their analysis in native brain tissue (Shariatgorji et al. 2014; Shariatgorji et al. 2016; Esteve et al. 2016).

DESI-MSI was employed over other techniques as it allows the direct mapping of the distribution and localisation of multiple molecular species in a single experiment (Shariatgorji et al. 2014; Shariatgorji et al. 2016). This is in contrast to the direct measurement of individual metabolite concentrations in brain tissue homogenates, which gives no information on distribution and localisation, and more traditional indirect methods like histological, immunochemical and ligand based assays (de Jong et al. 2005).

In addition plasma concentrations of homogentisic acid and tyrosine were measured using LC-MS/MS, to confirm that the murine model employed (1) had the expected biochemical phenotype observed in AKU (i.e. elevated HGA) and (2) hypertyrosinaemia was observed following treatment with nitisinone.

## Materials and methods

### Chemicals and reagents

Triethylamine (TEA), hydroxypropylmethylcellulose, polyvinylpyrrolidone and perchloric acid were purchased from Sigma-Aldrich (Dorset, UK). Water, methanol and trifluoroacetic acid (TFA) were obtained from Merck (Hohenbrunn, Germany). DPP-TFB was obtained from American Custom Chemicals Corporation (San Diego, CA, USA).

### Animal experiments

**Fig. 2:**
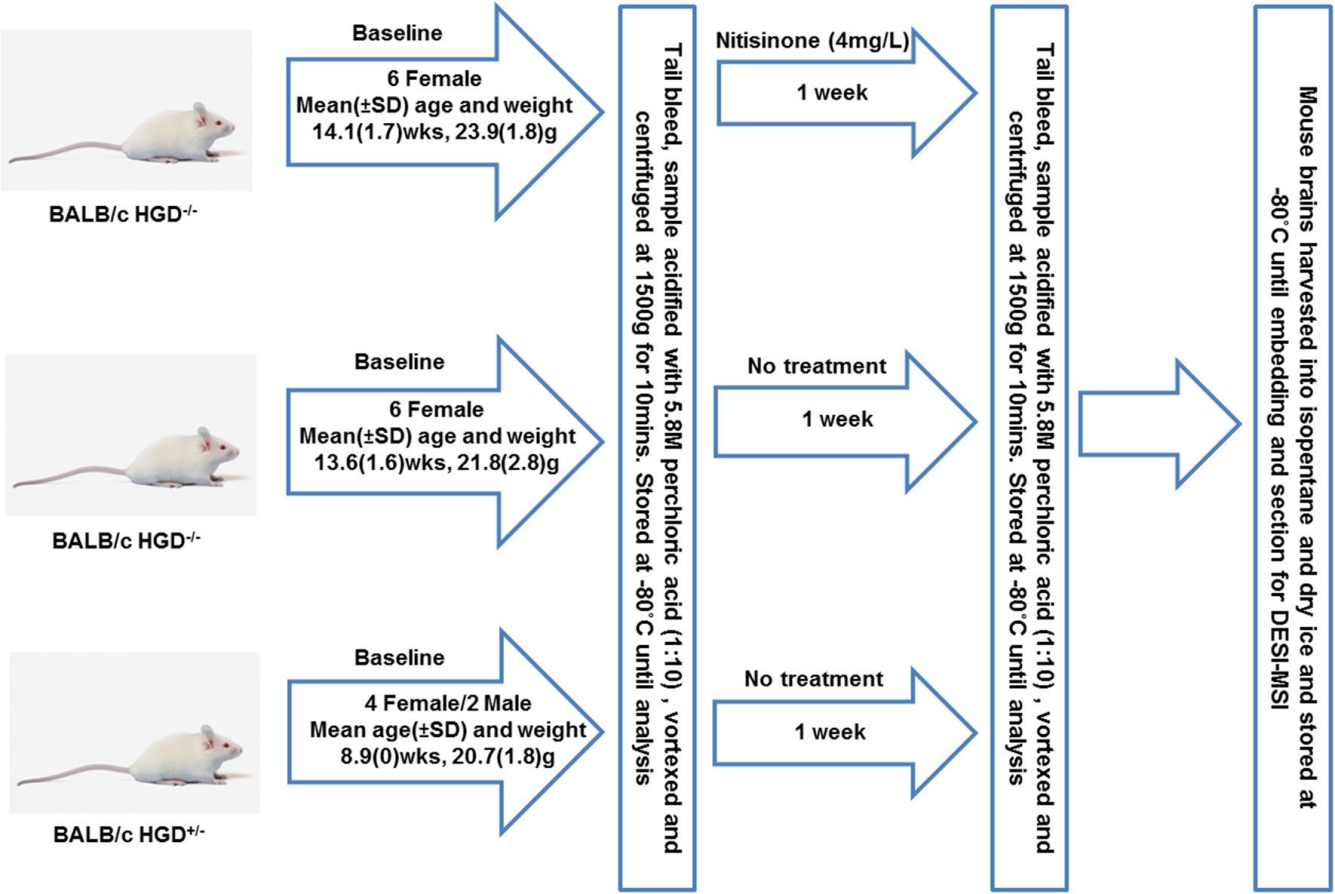
Summary of experimental workflow involving murine model of AKU, including mouse gender, weight and age; treatment groupings and sample collection, processing and storage. *SD* standard deviation

A murine model of AKU was used for all experiments as described previously (Preston et al. 2014). 18 BALB/c mice (Fig. 2) (12 HGD^−/−^ and 6 HGD^+/−^) were used in total. Six mice (HGD^−/−^) were administered nitisinone (4 mg/L) through drinking water for one week, the remaining 12 mice (6 HGD^−/−^ and 6 HGD^+/−^) received no nitisinone. All animals were housed in air conditioned rooms (with a 12hr dark/light cycle) at 20 °C and 53% humidity, with access to food and water *ad libitum* at the University of Liverpool. All animal experiments complied with the ARRIVE guidelines and were carried out in accordance with the U.K. Animals (Scientific Procedures) Act, 1986 and associated guidelines, EU Directive 2010/63/EU for animal experiments.

### Tail bleeds

Tail bleed samples were collected into Microvettes (Sarstedt, Germany) at baseline and after one week prior to culling. Whole blood samples were centrifuged at 1500*×g* for 10 mins at 4 °C and the plasma deproteinised by adding 60% 5.8 M perchloric acid (ratio 1:10, perchloric acid:plasma). Samples were vortexed and centrifuged at 1500×*g* for a further 10 mins. The supernatant was stored at − 80 °C until analysis (Fig. 2).

### Liquid chromatography tandem mass spectrometry analysis of mouse plasma

Plasma samples from tail bleeds were analysed for tyrosine, nitisinone and HGA using a previously published method (Hughes et al. 2015). In brief, samples were analysed for all three analytes in a single run on an Agilent 6490 triple quadrupole tandem mass spectrometer with Jet-Stream® electrospray ionization coupled with an Agilent 1290 Infinity II UHPLC pump. Separation was achieved on an Atlantis dC18 column (100 × 3.0 mm, 3 µm, Waters) maintained at 35 °C. Quantification was achieved using a matrix matched seven-point calibration curve and two product ion transitions for each analyte of interest (HGA 167 > 122 and 167 > 108, negative polarity; tyrosine 182 > 136 and 182 > 91; nitisinone 330 > 218, positive polarity).The linear measuring range for tyrosine, HGA and nitisinone were 60–2000; 15–500 and 0.5–10 μmol/L, respectively. 2 μL of deproteinized sample was diluted 1:1000 with 200 nmol/L ^13^C_6_-HGA, 500 nmol/L d_4_-tyrosine and 2 nmol/L ^13^C_6_-nitisinone in 0.1% formic acid/deionized water. 10μL of diluted plasma was injected onto the column. Data were acquired using MassHunter LC/MS Data Acquisition (version B.07.00, Build 7.0). HGA, tyrosine and nitisinone concentrations were calculated using MassHunter Quantitative Analysis (version B.06.00, Build 6.0).

### Brain harvesting

Brains were resected from each animal following cervical dislocation, and placed into a trough of dry ice cooled isopentane until any effervescence stopped. They were then removed using chilled forceps and wrapped in aluminium foil and stored at – 80 °C until dissection.

### Tissue processing and derivatisation

Frozen brain tissues were placed into plastic moulds and embedded into a matrix composed of 7.5% (w/v) hydroxypropylmethylcellulose and 2.5% (w/v) polyvinylpyrrolidone. Brains were divided into 3 moulds (n = 6 per mould, 2 brains from each treatment group) to allow axial (n = 6), sagittal (n = 6) and coronal (n = 6) sectioning to allow spatially resolved assessment of metabolite changes in brain substructures. This embedding approach was chosen in order to ensure that all brains are treated identically as it was previously shown that consecutive sectioning can lead to metabolite degradation (Swales et al. 2018). Although it was attempted to position brains into the moulds in exact same position and orientation, this approach might lead to slight differences in sectioning depth and position.

Embedded tissues were then cut using a cryo-microtome operated at − 20 °C (Leica CM3050S, Leica Microsystems, Wetzlar, Germany) to a thickness of 10 μm and thaw mounted onto superfrost microscope glass slides (Thermo Scientific, Braunschweig, Germany). These slides were subsequently dried under a gentle flow of nitrogen before being stored at – 80 °C until derivatisation and MSI analysis.

Brain tissues were derivatised as previously described (Shariatgorji et al. 2014; Shariatgorji et al. 2016) using DPP-TFB (9.2 mg DPP-TFB was dissolved in 7.2 mL of 75% methanol alkalinised with 3.5 μL of TEA to obtain a 1.3 mg/mL derivatisation solution). This was applied using a TM sprayer (HTX Technologies, Chapel Hill, NC) at 75 °C in 30 passes using criss-cross pattern [flow rate 80 μL/min, nitrogen flow of 10 psi, nozzle speed 1100 mm/min]. Chemical charge tagging of primary amine functional groups with DPP-TFB was done to enhance signal intensity of monoamine neurotransmitters as their analysis has proven difficult due to their low-ionization efficiency, spectral interferences from tissue components, ion suppression effects and analyte in source fragmentation (Shariatgorji et al. 2014; Shariatgorji et al. 2016; Esteve et al. 2016).

### Mass spectrometry imaging of mouse brain tissue

MSI data were acquired using an OmniSpray-2D DESI ion source (Prosolia Inc, Indianapolis, IN, USA) mounted onto a Q-Exactive Plus mass spectrometer (Thermo Scientific, Bremen, Germany). A home-built DESI sprayer assembly was used. This was based on a previously reported design (Takáts et al. 2004) that was modified as previously described (Abbassi-Ghadi et al. 2015). The distance between the sprayer and inlet capillary was 7 mm, the distance between sprayer and sample surface was 1.5 mm and the distance between inlet capillary and sample surface was < 1 mm. The angle between the sprayer and the sample surface was set to 75°, and the collection angle between inlet capillary and sample surface was 10°. Methanol/water (95:5 v/v) was used as the electrospray solvent, at a flow rate of 1.5 μL/min, and the spray voltage was set to + 4.5 kV. Solvent was delivered using a Dionex Ultimate3000 nLC pump (Sunnyvale, CA, USA). N4.8 Nitrogen (BOC, Guilldford, UK) was used as the nebulizing gas at a pressure of 7 bar. An S-Lens RF level of 75, capillary temperature of 320 °C and mass resolution of 70,000 were employed. Derivatised tissues were analysed in positive ion mode using a mass range of *m/z* 250–1000 and an injection time of 150 ms. All images were recorded with 100 µm spatial resolution resulting in a scan speed of 378.79 µm/s. Compound identities were assigned using exact mass only.

### Mass spectrometry imaging data analysis

MSI raw data files were initially converted into centroided .mzML files using MSConvert tool (ProteoWizard toolbox version 3.0.4043) and then further converted into .imzML format using imzML Converter version 1.3, RaceabIain et al. (2012), which were then converted to SCiLS data format for visualization and statistical analysis of the data in SCiLS Lab v2018b. (Bruker Daltonics, Bremen, Germany).

All *m/z* values for monoamine neurotransmitters were extracted with a mass window of less than 0.0052 Da. Derivatisation led to the formation of a [M + (DPP-TFB)]^+^ ion with no observable [M]^2+^ or [M + Na]^+^ ions. A peak corresponding to the DPP-TFB derivative was observed with a mass shift of +215.0855 to the exact monoisotopic mass. The differences between the theoretical and observed *m/z* values of each derivatised compound was < 5 ppm.

### Statistical analysis

A paired *t*-test was used to compare plasma HGA and tyrosine concentrations at baseline and 1 week using Graphpad Instat (version 3.10, 2009, CA, USA). A p value < 0.05 was deemed significant.

Mean intensity and standard deviation of raw *m/z* signal intensity for DPP-TFB derivatives of monoamine neurotransmitters were extracted from tissue regions of interest using SCiLS software (SCiLS Lab Bruker, version 2018b).To compare signal intensities for extracted *m/z* values for all compounds an unpaired t-test was used; a p value < 0.05 was deemed significant.

## Results

### LC-MS/MS analysis of plasma homogentisic acid and tyrosine

Plasma HGA concentrations were markedly higher in BALB/c HGD^−/−^ mice (AKU mice) at baseline compared to HGD^−/+^ mice (non-AKU mice). HGA concentrations in the HGD^+/−^ mice were below the lower limit of the measuring interval for the assay. Tyrosine concentrations were similar at baseline between the three groups studied; the variation observed between the three groups is thought to reflect dietary intake and normal variation in amino acid metabolism.

Treatment of HGD^−/−^ mice with nitisinone (mean ± standard deviation plasma concentration 0.4 ± (0.094) µmol/L) resulted in a marked decrease in HGA (9-fold, p = 0.004) and increase in tyrosine (9-fold, p = <0.0001) (Table 1).

**Table 1:** Plasma tyrosine and homogentisic acid concentrations at baseline and 1 week later in a murine model of AKU. *p < 0.05 deemed significant

Genotype	Nitisinone treated	Tyrosine (µmol/L, mean ± SD)			Homogentisic acid (µmol/L, mean ± SD)		
		Baseline	1-Week	p	Baseline	1-Week	p
HGD^−/−^ (n = 6)	Yes	112.3 (21.9)	997.8 (112.7)	<0.0001*	197.2 (87.2)	21.6 (4.3)	0.004*
HGD^−/−^ (n = 6)	No	92.8 (29.9)	77.6 (16.2)	0.096	215.7 (168.2)	158.2 (89.8)	0.18
HGD^+/−^ (n = 6)	No	66.5 (7.9)	62.6 (5.8)	0.27	<3.1	<3.1	NS

### Mass spectrometry imaging of neurotransmitters in murine brain tissue

**Fig. 3:**
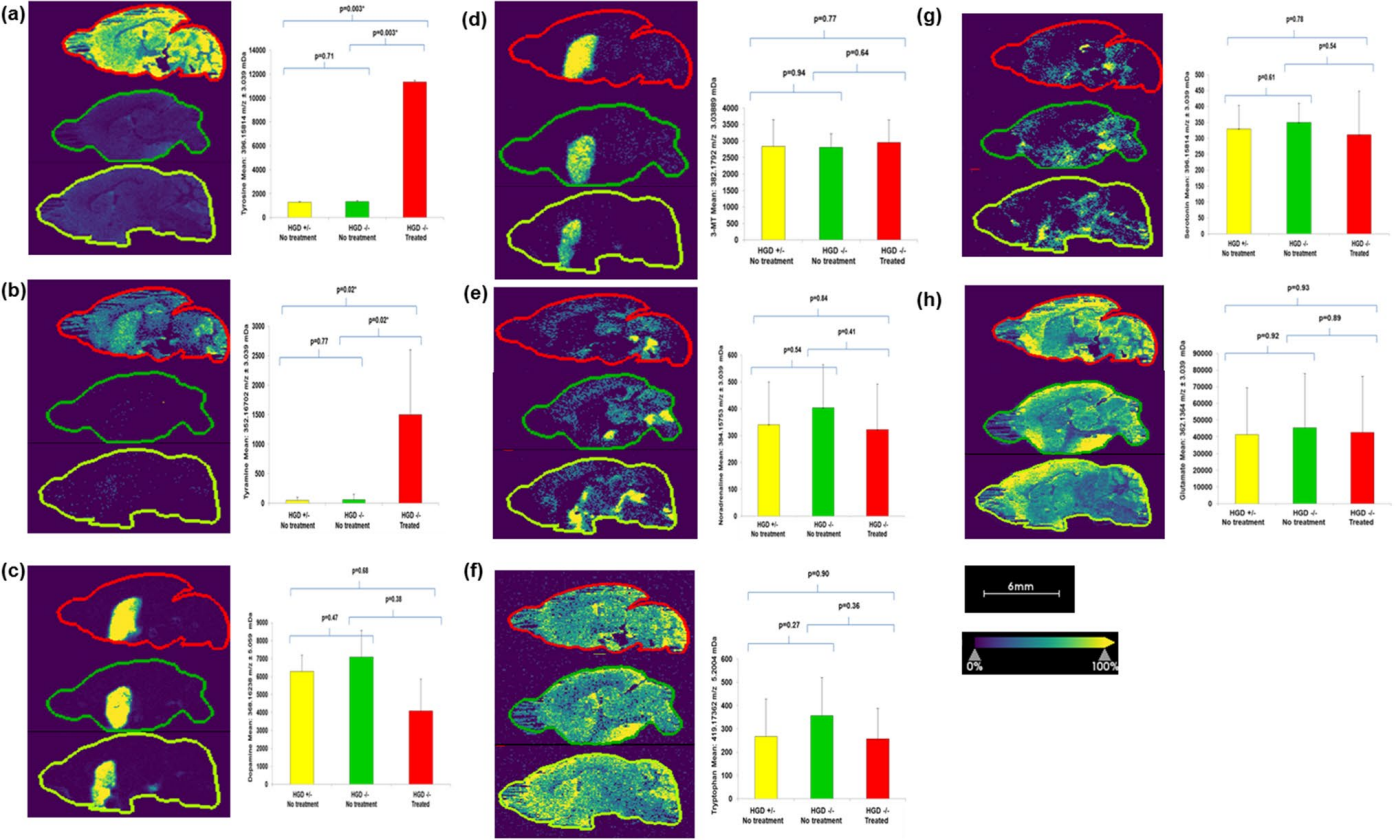
Representative mass spectrometry images of sagittal brain tissue sections from the BALB/c mouse. Ion intensity maps of precursor ions of DPP-TFB derivatives acquired in positive ionisation mode **a** tyrosine *m/z* 396.15814; **b** tyramine *m/z* 352.16702; **c** dopamine *m/z* 368.16238; **d** 3-MT *m/z* 382.18013; **e** noradrenaline *m/z* 384.15753; **f** tryptophan *m/z* 419.1753; **g** serotonin *m/z* 396.15854; **h** glutamate *m/z* 362.1364. In each panel *Left*: sagittal brain sections from BALB/c mice: HGD^−/−^ mouse treated with nitisinone (red outline); (2) HGD^−/−^ mouse no treatment (green outline) and (3) HGD^+/−^ no treatment (yellow outline). *Right*: bar graph of mean (± standard deviation) extracted *m/z* ion intensity for each group (n = 6 in each group); ion intensity for dopamine and 3-MT from striatum only, not the whole brain section. Scale bar: 6 mm; spatial resolution 100 μm. *p < 0.05 deemed significant. Signal intensities are indicated using a viridis colour scale 0–100% (blue to yellow)

MS images were successfully generated for all 18 murine brains included in this study in positive polarity. Differences in the appearance of sagittal images (Fig. 3) is a reflection of the positioning of brains’ in the mould. All brains were very similar in size and were anatomically symmetrical thus the plane sectioned for all six brains in each mould was considered to be comparable and thus ion intensities recorded were considered representative of the plane of tissue sectioned. Figure 3 shows representative sagittal MS images of BALB/c mice brains from all three treatment groups. As previously described (Shariatgorji et al. 2014; Shariatgorji et al. 2016; Esteve et al. 2016) chemical charge tagging of primary amines with DPP-TFB enabled the detection of dopamine, 3-MT, noradrenaline, serotonin, tyrosine, tryptophan and tyramine. It is important to note that noradrenaline has the same monoisotopic mass as 6-hydroxydopamine (*m/z* 169.0738), thus chemical charge tagging would also theoretically generate a mass shift of + 215.0855 to its exact monoisotopic mass. In this study it was assumed the mass shift observed at 384.1575 was noradrenaline. This is because it has been hypothesised that 6-hydroxydopamine is a neurotoxic metabolite of dopamine that is only observed in patients with Parkinson’s disease that are treated with L-DOPA (Borah and Mohanakumar 2012). It is proposed that 6-hydroxydopamine aggravates dopaminergic neurodegeneration, and it is this that forms the basis of its use in the generation of experimental models of Parkinson’s disease (Breese et al. 2005).

Glutamate was also detected, but it is important to note that tissue derivatisation is not required for this and native sections can be used (Shariatgorji et al. 2016). Using a derivatisation approach allowed analysis of all neurotransmitters in a single analytical run while the embedding approach ensured all tissues are treated identically at all times to minimise unequal degradation of neurotransmitters. Tissue drying was found to further increase stability (Swales et al. 2018).

Comparisons of *m/z* ion distribution maps for each compound were made between the three groups of mice: (1) BALB/c HGD^−/−^ (n = 6, no treatment) versus BALB/c HGD^−/−^ (n = 6, treated); (2) BALB/c HGD^+/−^ (n = 6, no treatment) versus BALB/c HGD^−/−^ (n = 6, treated); (3) BALB/c HGD^−/−^ (n = 6, no treatment) versus BALB/c HGD^+/−^ (n = 6, no treatment). It is important to note that comparisons made between *m/z* signal intensities represent a relative and not quantitative change in response. In agreement with previous authors (Shariatgorji et al. 2014; Shariatgorji et al. 2016) the total signal through the brain was used to assess spatial changes in neurotransmitter patterns, with the exception of dopamine and 3-MT in which only the signal relating to the striatum was used. This is because their production is very localised and specific to this anatomical region of the brain. In contrast the other neurotransmitters and related metabolites are distributed more ubiquitously throughout the brain and so the total brain area was used.

Tyrosine (10-fold, p = 0.003; 10 fold, p = 0.003; and p = 0.71) and tyramine (25 fold, p = 0.02; 32 fold, p = 0.02; p = 0.77) showed significantly more intense signals in mice that received treatment with nitisinone (Fig. 3). Signal intensities for tyrosine and tyramine increased in all regions of the brains studied. Signals were most intense in the *cerebellum*, *cerebral cortex* and *hypothalamus* for tyrosine, and the *striatum* and *cerebellum* for tyramine. No differences in ion distribution or intensity were observed for the two groups of mice that did not receive nitisinone treatment.

Dopamine (striatum of brain only, p = 0.38, p = 0.68, and p = 0.47, respectively) and its metabolites 3-MT (striatum of brain only, p = 0.67, p = 0.77, and p = 0.94, respectively) and noradrenaline (p = 0.41, p = 0.81, and p = 0.54, respectively) did not change significantly following treatment with nitisinone. The intensity of the signal for dopamine and 3-MT was highest in the *striatum*, *cortical subplate*, and *palladium* (Fig. 3). The intensity of the signal for noradrenaline was more dispersed than that of dopamine and 3-MT, and was most intense in the *substantia nigra reticulata* and *ventral striatum*.

Serotonin (p = 0.54, p = 0.78, and p = 0.64, respectively) and its precursor amino acid tryptophan (p = 0.36, p = 0.90, and p = 0.27, respectively), and glutamate (p = 0.89, p = 0.93, and p = 0.92, respectively) showed no significant differences between mice that received nitisinone and those that did not. The signal intensity for serotonin was most intense in the *substantia nigra reticulata* and *ventral striatum*, while its precursor tryptophan and glutamate were found in all brain tissues. The latter being most intense in the *cerebral cortex* (Fig. 3).

All monoamine neurotransmitter compounds and related metabolites in the two groups of mice that did not receive nitisinone treatment were not significantly different (Fig. 3).

## Discussion

To date the direct consequences of nitisinone induced hypertyrosinaemia on neurotransmitter metabolism in patients with AKU has not been reported. Herein for the first time we report the direct measurement of DPP-TFB derivatives of monoamine neurotransmitters in the brain tissue from a murine model of AKU following treatment with nitisinone, and corresponding plasma concentrations of HGA and tyrosine.

### LC-MS/MS analysis of plasma homogentisic acid and tyrosine

Plasma HGA concentrations were markedly higher in BALB/c HGD^−/−^ mice (AKU mice) at baseline compared to HGD^−/+^ mice (non-AKU mice), this was expected and entirely in keeping with the genotype of the mice studied (Preston et al. 2014).

Treatment of BALB/c HGD^−/−^ mice with nitisinone resulted in an expected decrease in HGA and increase in tyrosine. The changes observed occurred because nitisinone is reversible competitive inhibitor of *HPPD* (Fig. 1). These finding confirm that the mice used in this study had the biochemical phenotype one would expect in AKU and that hypertyrosinaemia was observed following treatment with nitisinone.

### Mass spectrometry imaging of neurotransmitters in murine brain tissue

Tyrosine and tyramine increased significantly in BALB/c HGD^−/−^ mice treated with nitisinone. No changes in tyrosine or tyramine were observed in HGD^−/−^ and HGD^−/+^ mice that received no treatment. These novel findings support that the changes observed were a consequence of treatment with nitisinone. The increase in tyrosine is likely to be the result of the 9-fold increase in plasma tyrosine, which through facilitated diffusion via the LAT-1 transporter (Mastroberardino et al. 1998) will have resulted in increased transport into the central nervous system. The large increase in tyramine was an unexpected finding, and is a likely consequence of the action of aromatic acid decarboxylase, which converts tyrosine to tyramine. The physiological role and mechanisms of action of this trace amine remain poorly understood in mammals. Tyramine is often regarded as a by-product of amino acid metabolism with no clear functional relevance (Ledonne et al. 2011).

It is reassuring that dopamine did not increase itself and is likely to reflect that its synthesis is highly regulated. There is a major and minor route for dopamine biosynthesis (Meiser et al. 2013). The minor route involves the enzymatic conversion of tyrosine to tyramine via the action of aromatic acid decarboxyalse, tyramine is then converted to dopamine via cytochrome CYP2D6. The major route is via the enzymatic conversion of tyrosine to L-DOPA and then to dopamine via the actions of tyrosine hydroxylase and then aromatic acid decarboxylase. In the face of excess tyrosine and tyramine and no change in dopamine one may postulate that one or all of the enzymes mentioned are down regulated to limit dopamine production when it is not required. A limitation of this study is that enzyme activity was not measured. The increased catabolism of dopamine to noradrenaline via the enzymatic action of dopamine hydroxylase, or to 3-MT via the enzymatic action of catechol-*o*-methyltransferase is unlikely as no significant changes were observed in either of these compounds following treatment with nitisinone (Fig. 3).

Importantly this study did not show any changes in serotonin or tryptophan metabolism in HGD^−/−^ mice following treatment with nitisinone, confirming that hypertyrosinaemia does not affect serotonin biosynthesis or metabolism in the murine model of AKU studied.

In addition it is reassuring that no significant changes were observed in neurotransmitters or their metabolites at baseline and 1 week in the HGD^−/−^ and HGD^−/+^ mice that received no treatment, supporting that changes that were observed were a consequence of treatment with nitisinone.

Whilst there have been no reported investigations of the impact of nitisinone induced hypertyrosinaemia on dopaminergic and serotoninergic neurotransmitter metabolism in AKU, the impact of nitisinone induced hypertyrosinaemia on neurotransmitter metabolism has been investigated in a mouse model of phenylketonuria (PKU) (Harding et al. 2014) and HT1 (Hillgartner et al. 2016).

Hillgartner et al. (2016) used a similar dose of nitisinone to our group and reported that urinary homovanillic acid (dopamine metabolite) increased 4-fold following nitisinone, but concluded that this was likely to be a reflection of peripheral breakdown and metabolism and did not reflect changes in the central nervous system. In this study no direct measurements of dopamine or serotonin were made in brain tissue, but they concluded that slower learning and cognitive difference in the mice studied were caused by HT1 and not by the treatment with nitisinone. We have also reported an increase in a urinary dopamine metabolite (3-MT) following nitisinone treatment in AKU patients (Davison et al. 2018a, b) and are in agreement that this is likely to reflect a change in peripheral metabolism of catecholamines.

In contrast Harding et al. (2014) used a much higher dose of nitisinone (4 mg/mL) than our group (4 mg/L) and as expected this led to hypertyrosinaemia; a six-fold increase in mean serum tyrosine (256 μmol/L), and a slight but, significant increase in brain tyrosine was observed. This was however far less than what we observed (9-fold increase in mean plasma tyrosine [mean tyrosine 997 µmol/L] and 10-fold increase in tyrosine in the brain tissue) on a thousand times lower dose of nitisinone. This can be explained in large by the metabolic defect in the tyrosine metabolic pathway being proximal to the site of nitisinone action in PKU and distal in AKU (Fig. 1). In PKU, phenylalanine cannot be converted to tyrosine and thus far less accumulates as a consequence of nitisinone inhibiting *HPPD*, in large the observed increase will reflect the accumulation of dietary tyrosine.

Harding et al. (2014) also observed that phenylalanine decreased by 44% and dopamine increased by 36% in brain tissue following nitisinone treatment. Herein no significant changes in dopamine in brain from the AKU mouse were observed following nitisinone. It has been proposed that the lower phenylalanine concentration in brain tissue reduced phenylalanine mediated inhibition of tyrosine hydroxylase, thus enabling dopamine biosynthesis (Harding et al. 2014). These differences are likely to be accounted for by the fact that Harding et al. (2014) performed analysis in brain tissue from a mouse model of PKU and not AKU, which have different metabolic defects.

Importantly in the AKU mouse model there was no significant difference in brain serotonin in HGD^−/−^ mice following treatment with nitisinone, or between HGD^−/−^ and HGD^+/−^ that received no treatment. Serotonin concentrations did not change in PKU animal model following treatment with nitisinone either, but did remain depressed in the PKU mice whether they were treated or not, compared to wild type mice (Harding et al. 2014). It is thought that this was caused by phenylalanine inhibiting the enzyme tryptophan hydroxylase, which regulates the rate limiting step of serotonin metabolism, and cannot be recovered by nitisinone treatment (Harding et al. 2014). Recently Winn et al. (2018) provided further support for this hypothesis in a study that showed that while blood phenylalanine reduction corrects dopamine and serotonin deficiencies in the central nervous system; it did not recover the activity of tryptophan hydroxylase.

Herein we also showed that glutamate does not change following treatment with nitisinone. The reason for measuring this neurotransmitter was that the glutaminergic neurotransmitter system is known to have a close relationship with dopaminergic and serotoninergic systems (Andreou et al. 2015). Dysregulation of either of the latter may have resulted in a compensatory change in the glutaminergic system.

## Conclusions

Treatment with nitisinone did not affect the ion distribution or intensity of the neurotransmitters dopamine, noradrenaline and serotonin in the AKU mouse brain, suggesting that their concentrations do not change. Neurotransmitter precursor’s tyrosine and tyramine were significantly increased following treatment with nitisinone. The significance and fate of these metabolites is unknown and requires further investigation. These findings have critical importance for the use of nitisinone in patients with AKU as they clearly demonstrate that nitisinone does not have a direct effect on monoamine neurotransmitter metabolism in the central nervous system of mice with AKU. If these findings translate to patients with AKU treated with nitisinone, one can postulate that the hypertyrosinaemia occurring following treatment with nitisinone does not affect monoamine neurotransmitter metabolism and thus is unlikely to result in altered mood or cognition.

## Abbreviations

AKU: Alkaptonuria
HGA: Homogentisic acid
*HGD*: Homogentisate-1,2-dioxygenase
*HPPD*: Hydroxyphenylpyruvic acid dioxygenase
HT1: Hereditary Tyrosinaemia Type 1
DESI: Desorption electrospray ionisation mass spectrometry imaging
MSI: Mass spectrometry imaging
LC-MS/MS: Liquid chromatography tandem mass spectrometry
DPP-TFB: 2,4-diphenyl-pyranylium tetrafluoroborate
TEA: Triethylamine
TFA: Trifluoroacetic acid
PKU: Phenylketonuria
3-MT: 3-methoxytyramine
