# Correction to: Assessing the effect of nitisinone induced hypertyrosinaemia on monoamine neurotransmitters in brain tissue from a murine model of alkaptonuria using mass spectrometry imaging

**DOI:** 10.1007/s11306-019-1540-3

**Published:** 2019-05-18

**Authors:** Andrew S. Davison, N. Strittmatter, H. Sutherland, A. T. Hughes, J. Hughes, G. Bou-Gharios, A. M. Milan, R. J. A. Goodwin, L. R. Ranganath, J. A. Gallagher

**Affiliations:** 1 Department of Clinical Biochemistry and Metabolic Medicine, Liverpool Clinical Laboratories, Royal Liverpool University Hospitals Trust, Liverpool, L7 8XP UK; 2 Musculoskeletal Biology I, Institute of Ageing and Chronic Disease, University of Liverpool, Liverpool Health Partners, Liverpool, UK; 3 Pathology, Drug Safety and Metabolism, IMED Biotech Unit, AstraZeneca, Cambridge, UK

## Correction to: Metabolomics (2019) 15:68 10.1007/s11306-019-1531-4

The original publication of this article contained an incorrect version that did not include some final reviewers' suggestions, was inadvertently received for production and published.

The original article has been corrected.

